# The Critical Role of Lambda-Shaped QRS-ST-T Wave Fusion ST-Elevation in Diagnosing and Managing Takotsubo Cardiomyopathy: A Case Report and Review of Literature

**DOI:** 10.7759/cureus.49037

**Published:** 2023-11-19

**Authors:** Victor H Molina-Lopez, Jose Escabi-Mendoza

**Affiliations:** 1 Department of Cardiology, Veterans Affairs (VA) Caribbean Healthcare System, San Juan, PRI

**Keywords:** shark fin st-elevation ecg sign, takotsubo cardiomyopathy, triangular qrs-st-t wave st-elevation, stress cardiomyopathy, stress-induced cardiomyopathy

## Abstract

Stress-induced cardiomyopathy, commonly known as Takotsubo cardiomyopathy (TCM), is a clinical syndrome characterized by acute and transient ventricular systolic dysfunction that often presents with chest pain and may resemble an acute coronary syndrome. This case report discusses a complex clinical scenario involving an adult female with severe depression who attempted suicide through drug overdose, subsequently developing serotonin syndrome. Her clinical presentation was further complicated by the emergence of a unique lambda-shaped triangular QRS-ST-T waveform fusion ST-elevation electrocardiographic (ECG) pattern closely mimicking an anterolateral occlusive myocardial infarction. The study delves into the clinical implications of this unique ECG pattern in TCM, providing valuable insights into diagnosing and treating such complex cases. This case underscores the importance of recognizing diverse manifestations of TCM and its potential for severe cardiovascular complications.

## Introduction

Stress-induced cardiomyopathy, commonly recognized as Takotsubo cardiomyopathy (TCM) or "broken heart syndrome", is a transient form of acute systolic dysfunction primarily induced by acute emotional or physiological stressors [1-7]. It is a diagnosis of exclusion and presents a notable challenge due to its frequent manifestation with chest pain and electrocardiographic (ECG) findings that mimic a true acute coronary syndrome (ACS) [1-7]. The mechanism of TCM involves an excessive release of catecholamines, leading to microvascular perfusion impairment, myocardial inflammation, and electrophysiological changes [1-7]. 

In this report, we describe the case of an adult female with a history of severe depression who attempted suicide through an intentional drug overdose with citalopram, subsequently developing serotonin syndrome. The clinical presentation was further complicated by the emergence of acute chest pain and a distinct lambda-shaped ST-elevation ECG pattern characterized by triangular QRS-ST-T waveform fusion, closely resembling an occlusive myocardial infarction (OMI). The patient subsequently developed an acute tachyarrhythmia due to supraventricular tachycardia (SVT) as well as cardiogenic shock requiring vasopressor support and mechanical circulatory support (MCS). The study delves into the clinical implications of this unique ECG pattern in TCM, providing valuable insights into diagnosing and treating such complex cases.

## Case presentation

A 65-year-old female with a medical history of hypertension, hypercholesterolemia, and severe depression was brought to the emergency department by her concerned family. The patient's family reported that she had been experiencing symptoms of nausea, vomiting, diarrhea, and anorexia over the previous day. Upon initial assessment, vital signs indicated tachycardia at 120 beats per minute, a blood pressure of 120/80 mmHg, and a respiratory rate of 25 breaths per minute. There was no fever noted. During the physical examination at the emergency room (ER), the patient appeared restless, disheveled, anxious, and agitated, with moist skin and a flushed complexion. Dry mucous membranes and a fine tremor in her outstretched hands were observed. Pupils were dilated, and ocular nystagmus was noted. Abdominal examination revealed increased bowel sounds. The neurological examination showed clonus in the lower extremities, hyperreflexia, and bilateral Babinski signs.

**Figure 1:**
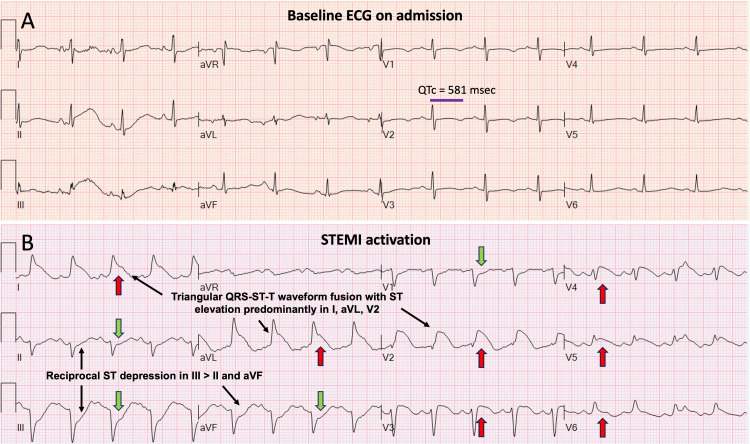
ECG evolution from the time of admission to the episode of acute chest pain and STEMI code activation. (A) Baseline ECG with sinus rhythm, nonspecific ST changes, and a prolonged QTc (purple line) at time of admission. (B) ECG during acute episode of chest pain revealed sinus rhythm with ST elevation (red arrows) in leads I, aVL, and V2-V6, with reciprocal ST depression and T wave inversion in the inferior leads (green arrows). The lambda-shaped triangular morphology resulting from the fusion of the QRS-ST-T waveforms raises the concern for an anterolateral OMI with likely LM or LAD culprit high-risk lesion with a large burden of myocardial ischemia. Interestingly, the inferior leads have reciprocal ST depressions that resemble reciprocal lambda-shaped QRS-ST-T wave fusion morphology. ECG: Electrocardiogram; STEMI: ST-elevation myocardial infarction; LM: left main coronary artery; LAD: left anterior descending artery; OMI: occlusive myocardial infarction.

Laboratory evaluation revealed mild hypomagnesemia and hypokalemia promptly addressed through intravenous (IV) replacement therapy. A 12-lead ECG performed upon initial evaluation showed normal sinus rhythm with a prolonged QTc interval (Figure 1A). Chest radiography performed at admission did not reveal any significant abnormalities. The patient's home medication regimen included citalopram 20 mg once daily, buspirone 20mg twice daily, atorvastatin 20mg once daily, and losartan 25 mg once daily. Acute serotonin syndrome was suspected due to acute selective serotonin reuptake inhibitor (SSRI) intoxication from intentional overdose. Consequently, she was admitted to the hospital for acute medical and psychiatric care. While admitted, she displayed progressively worsening depressive mood accompanied by suicidal ideation, necessitating constant observation, intensive psychiatric care, and physical restraints to prevent self-harm. On the second day of her hospitalization, the patient reported experiencing severe oppressive retrosternal chest pain accompanied by diaphoresis. The ECG revealed ST elevation in anterolateral and high lateral leads with a lambda-shaped triangular QRS-ST-T waveform fusion (Figure 1B).

A bedside ultrasound raised concerns about severe anteroseptal and septal hypokinesis of the segments with severe LV dysfunction. Her vital signs were notable for a heart rate of 110 beats per minute, a blood pressure of 110/60 mmHg, and a respiratory rate of 20 breaths per minute. The chest pain was rated as 8/10 of intensity and decreased to 5/10 after administration of sublingual nitroglycerin. IV heparin and chewable aspirin (325 mg) were administered, and an ST-elevation myocardial infarction (STEMI) code was activated to facilitate emergent coronary angiography. There were no acute electrolyte disorders, with normal serum magnesium and potassium levels.

**Figure 2:**
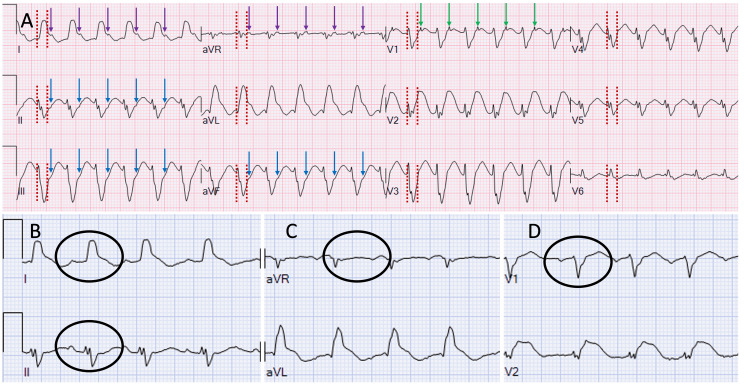
SVT during acute chest pain event and after synchronized cardioversion. (A) ECG with SVT at ~ 150 bpm with a QRS width of 120 ms (red dashed lines), with retrograde P waves following each QRS complex suggesting a fast-slow re-entry tachycardia. Note the upright P waves in V1 with the appearance of pseudo R' waves (green arrow). Inverted P waves can be seen in II, III, and aVF (blue arrows). Upright retrograde P waves can also be appreciated in I and aVR (purple arrows). The retrograde P waves resolved on the resolution of arrhythmia, as seen in leads I and II, aVR, and V1 (B-D). The persistence of the baseline QRS axis and morphology was helpful to distinguish the SVT from ventricular tachycardia. Patients with a triangular QRS-ST-T waveform fusion can pose a significant challenge when evaluating for malignant arrhythmias. When there is a preexisting conduction abnormality, an SVT can appear to have a wide QRS complex, but the morphology and axis should be identical to the sinus QRS – as seen in this case when the morphology of (A) is compared with (B-D). In cases of rate-related aberrant conduction, the QRS can also appear widened. ECG: Electrocardiogram; SVT: supraventricular tachycardia; bpm: beats per minute

However, the patient's clinical presentation deteriorated significantly during preparation and transit to the cardiac catheterization suite. Vital signs revealed a pulse rate increase to 150 beats per minute, her blood pressure of 80/40 mmHg (mean arterial pressure (MAP) 53 mmHg), respiratory rate of 40 breaths per minute, and peripheral oxygen saturation of 85% on room air. She became agitated and diaphoretic and was unresponsive to verbal commands. She was endotracheally intubated and mechanically ventilated with a fraction of inspired oxygen (FiO_2_) of 100%, tidal volume of 8 mL/kg ideal body weight, set respirations at 18 breaths/min, and positive end-expiratory pressure (PEEP) of 5 cm H_2_O. Her core temperature was 36°C. Her physical examination revealed a normal S1 and S2, no S3 or S4, and no audible murmur. Jugular veins were distended to the level of the mandible, and lung fields revealed crackles. Extremities were cool to the touch with sluggish capillary refill. Tachycardia was attributed to SVT with fast-slow atrioventricular re-entry tachycardia mechanism, which was challenging to identify promptly due to the Lambda-shaped morphology from the QRS-QT-T wave fusion (Figure 2A). Management involved synchronized cardioversion due to hemodynamic instability, eventually converting to sinus rhythm after cardioversion (Figures 2B-2D). The SVT likely precipitated the acute hemodynamic collapse while in transit to the angiography suite.

**Figure 3:**
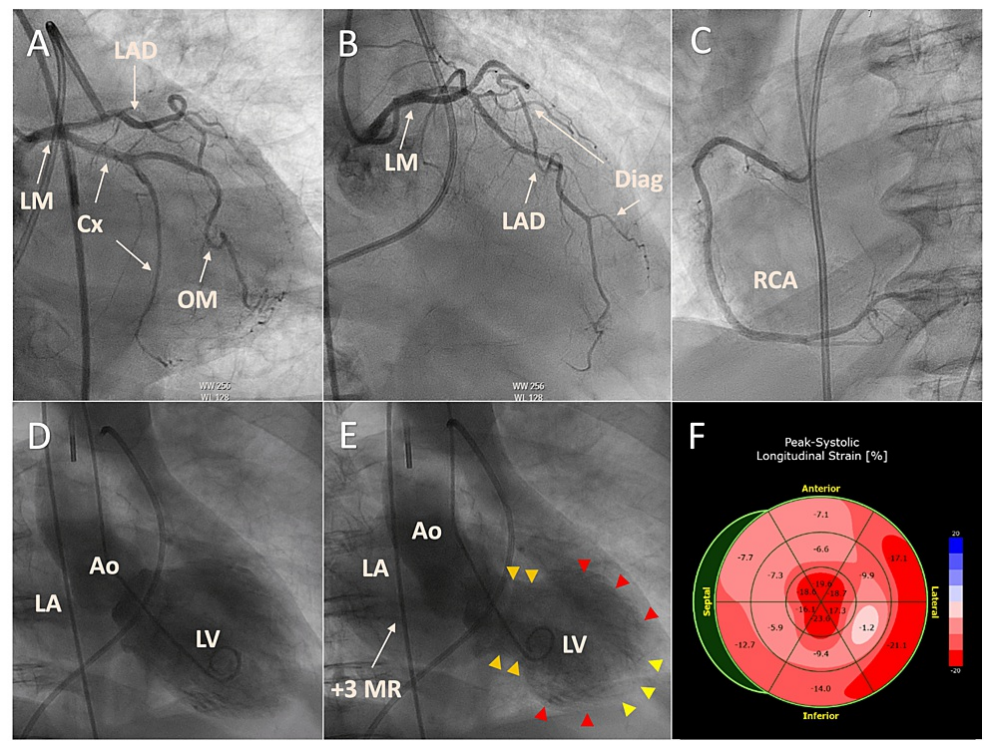
Emergent coronary angiography and ventriculography. Emergent coronary angiography revealed the absence of obstructive coronary artery disease (A-C). Ventriculography revealed severe LV dysfunction with LVEF of 20-25% with predominant mid-ventricular wall akinesis (red arrowheads), sparing of the apex (yellow arrowheads), and hyperkinesis of the anterobasal and inferobasal segments (orange arrowheads). An associated moderate-to-severe (Seller's grade +3) acute MR can be appreciated on ventriculography (E). Strain polar plot (F) by TTE speckle-tracking revealed decreased longitudinal strain affecting predominantly the mid-ventricular segments and sparing the apical and basal segments. However, the anterior and anteroseptal basal and mid segments have decreased longitudinal strain, consistent with myocardial injury of the LAD territory distribution. This strain analysis is consistent with a large burden of myocardial mass that correlates with the large anterolateral myocardial injury pattern suggested by the ECG. LM: Left main coronary artery; LCx: left circumflex coronary artery; OM: obtuse marginal coronary artery branches; LAD: left anterior descending coronary artery; RCA: right coronary artery; Ao: aorta; LV: left ventricle; LA: left atrium; MR: mitral regurgitation; Diag: diagonal coronary artery branches; LVEF: left ventricular ejection fraction; TTE: transthoracic echocardiography

Emergent coronary angiography revealed the absence of obstructive coronary artery disease (Figures 3A-3C). The left ventriculogram demonstrated severe LV dysfunction with a left ventricular ejection fraction (LVEF) of 20-25% with predominant mid-ventricular wall akinesis, sparing of the apex, and hyperkinesis of the anterobasal and inferobasal segments (Figures 3D, 3E). An associated moderate-to-severe (+3) acute MR can be appreciated on ventriculography (Figure 3E). The left ventricular end-diastolic pressure measured 45 mmHg. Right heart hemodynamics were consistent with acute pulmonary edema due to acute left ventricular systolic dysfunction, acute MR, and cardiogenic shock. Large V waves were present in the pulmonary wedge pressure tracings. Bedside echocardiography revealed no left ventricular outflow tract obstruction (LVOTO) or systolic anterior motion of the mitral valve (SAM). MCS with an intra-aortic balloon pump (IABP) was initiated alongside high-dose vasopressor support with norepinephrine and epinephrine with subsequent improvement in cardiac output.

**Figure 4:**
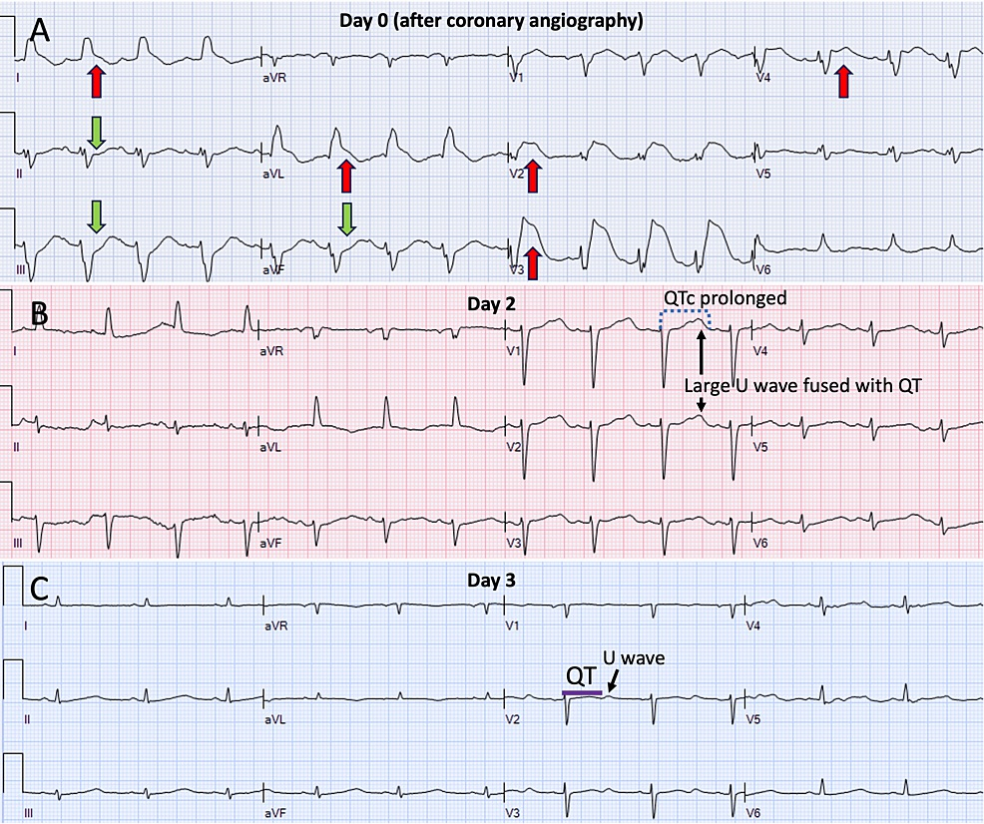
ECG evolution after acute event (day 0) to day 3. As the patient clinically improved, the ST elevations and QRS widening improved (A-C) without forming pathological Q-waves. ECG: Electrocardiogram

Highly sensitive troponin-T levels were markedly elevated in the range of 600-700 ng/L (abnormal cutoff > 13 ng/L). These findings established a diagnosis of a predominantly mid-ventricular TCM variant triggered by severe depression and serotonin syndrome, with hemodynamic collapse precipitated by the onset of arrhythmia. Over the subsequent days, ECG changes continued to normalize, with resolution of the ST elevations without developing new Q waves (Figures 4A-4C). The cardiogenic shock was managed with supportive therapy, and the patient was gradually weaned from the IABP, mechanical ventilator, and vasopressor therapy, subsequently transitioning to guideline-directed medical therapy for heart failure with reduced ejection fraction as her condition improved. The patient was initially maintained on IV heparin while on IABP, then transitioned to oral anticoagulation due to extensive LV hypokinesis. Echocardiography documented an improvement in LVEF from 20-25% acutely to 45-50% at two weeks and 55-60% at two months of follow-up.

## Discussion

TCM manifests as a sudden, typically reversible left ventricular (LV) dysfunction, predominantly affecting women and often triggered by stressful emotional or physical events [1-7]. Drug-induced TCM, a form of physical stress, mainly results from either direct (exogenous) stimulation by catecholamines or indirect stimulation through agents like catecholamine reuptake inhibitors, sympathomimetics, anaphylaxis, or a hyperadrenergic state due to drug withdrawal [1-7]. In our case, the patient experiencing severe depression and medication overdose developed serotonin syndrome, a condition caused by medications that heightened serotonergic activity. This syndrome is characterized by a clinical triad of altered mental state and autonomic and neuromuscular hyperactivity, which can lead to a hyperadrenergic state and increase the risk of adverse cardiovascular events, including TCM. 

TCM is typically diagnosed in patients suspected of having ACS when cardiac catheterization reveals no coronary obstruction [1-7]. It is found in 0.7-2.5% of such patients, with the majority (90.7%) being women between 62 and 76 years [4]. Common symptoms during onset include chest pain (83.4%) and shortness of breath (20.4%), usually triggered by emotional or physical stressors [4]. On admission, ST-segment elevations can be seen in the ECGs of 71.1% of these patients, while about 85.0% show a slight elevation in Troponin levels [4]. The updated Mayo Clinic criteria for TCM diagnosis encompass transient LV mid-segment dyskinesia, wall motion irregularities surpassing a single epicardial distribution, lack of obstructive coronary disease or plaque rupture, new ECG anomalies (like ST-elevations, ST-depressions, T-wave inversions, or QTc prolongation) or a minor troponin elevation, and ruling out conditions like pheochromocytoma and myocarditis [5]. The InterTAK Diagnostic Score gauges the probability of TCM's occurrence and distinguishes it from ACS with a high degree of accuracy [6,7].

Patients with TCM have four distinct phenotypes based on LV wall motion abnormalities [8]. The apical variant (classic) is the most common, making up 70-80% of cases and featuring apical akinesis and basal hypercontractility [8-10]. Atypical variants can be midventricular (10-15%), basal or "reverse" (2-20%), and focal (< 2%) [8-10]. Except for the focal type, wall motion abnormalities are not restricted to a single coronary artery territory [8]. About 25% of cases involve the right ventricle and are considered a marker for a more severe clinical course and worse outcomes [11,12]. 

A vital aspect of the case is the lambda-shaped triangular QRS-ST-T wave fusion pattern that resembled an OMI affecting the LM or proximal LAD coronary artery, indicative of a high-risk ACS. In STEMI cases, the triangular QRS-ST-T fusion waveform pattern on an ECG typically indicates involvement of the LM and a large ischemic burden [13]. This ECG pattern is associated with an increased risk of ventricular fibrillation, cardiogenic shock, and higher in-hospital mortality rates, especially compared to patients with other types of ST-segment elevation on their ECGs [13]. It is also associated with hyper-acute STEMI and is considered a sign of impending hemodynamic instability, necessitating rapid and intensive medical intervention for patient survival [14]. In TCM patients, the emergence of a triangular QRS-ST-T waveform pattern on an ECG is rare (3.2%), but when it occurs, it is linked to more severe physical stress, more severe ventricular dysfunction, higher risk of complications during hospitalization, and worse long-term outcomes in up to 80% of these cases [15,16]. This ECG pattern may arise regardless of a culprit coronary artery if a significant burden myocardial injury is present [15,16]. Factors that may predict an adverse in-hospital outcome include the presence of a physical trigger, acute neurological or psychiatric conditions, initial troponin levels greater than ten times the upper reference limit, and an admission LVEF below 45% [17].

While in transit to the catheterization laboratory, the patient developed tachycardia with subsequent hemodynamic collapse, cardiogenic shock, and respiratory failure. Initial ECG tracings showed wide complex tachycardia, but retrograde P waves following each QRS complex suggested a fast-slow re-entry tachycardia mechanism (Figures 2A-2D). Patients with a triangular QRS-ST-T waveform fusion pattern can pose a significant challenge while evaluating for malignant arrhythmias. When there is a preexisting conduction abnormality, an SVT can appear to have a wide QRS complex, but the morphology and axis should be identical to the sinus QRS, as seen in Figures 2A-2D. TCM has been correlated with various cardiac arrhythmias, including life-threatening atrioventricular block, ventricular tachycardia, and ventricular fibrillation [7]. Among the common cardiac tachyarrhythmias, atrial fibrillation occurs in 6.9-15.7% of patients, followed by ventricular tachycardia (3.2%), atrial flutter (1.9%), ventricular fibrillation and flutter (1%), and paroxysmal supraventricular tachycardia (0.8%) [18-20]. The condition is often accompanied by repolarization anomalies, manifesting as distinct T-wave abnormalities and QT interval prolongation. These electrophysiological alterations predispose individuals to an elevated likelihood of experiencing ventricular arrhythmias, including Torsades de pointes [7,18-20]. About 2% can experience sudden cardiac arrest [19]. Patients with arrhythmias, especially atrial fibrillation, may have more extended hospital stays, higher patient care costs, and increased mortality [18-20]. 

Treatment involves inpatient hospital care with hemodynamic and telemetry monitoring and supportive care until LV function improves, usually within 21 days after onset [7,20]. In hemodynamically stable patients, treatment may encompass diuretics and vasodilators to alleviate pulmonary congestion, along with beta-blockers and angiotensin-converting enzyme inhibitors or angiotensin II receptor blockers to reduce workload and manage hypertension [7,20]. An echocardiogram is recommended for patients with unstable hemodynamics to assess the presence of LVOTO [7]. Beta-blockers may reduce basal contractility and alleviate the LVOTO [7]. For patients without LVOTO, inotropic medications like milrinone, dobutamine, and dopamine are potential treatment options [7]. LVOTO may contraindicate the use of inotropic medications and IABP. Inotropes increase basal hypercontractility and may worsen the LVOTO. If LVOTO is present, initial medical interventions are limited to fluid resuscitation, vasopressors, and beta-blockers [20]. In those cases, vasopressors, MCS with left ventricular assist devices, or extracorporeal membrane oxygenation may be required [13]. Anticoagulation can be considered a measure to reduce the risk of thromboembolic stroke in patients with severe LV dysfunction and hypokinesis [20]. Patient care involves serial echocardiography for recovery monitoring [7,20].

In this case, respiratory failure was likely due to cardiogenic shock and acute mitral insufficiency, which may occur in cases with hyperdynamic basal segments, SAM of the mitral valve, or due to poor mitral valve coaptation. The onset of an SVT further precipitated the acute deterioration into respiratory failure and hemodynamic collapse. Our case exemplifies the importance of bedside echocardiographic evaluation for LVOTO, as this step is crucial for guiding hemodynamic supportive therapy acutely. While LVOTO was not present by echocardiography, this patient was not provided inotropic support to avoid increasing basal hyperkinesia. If MCS is considered, IABP may be a cost-effective option in resource-limited settings to treat severe cardiogenic shock if no LVOTO is identified by bedside echocardiography, even if acute mitral insufficiency ensues during the acute phase. Our patient was provided anticoagulation during the acute phase without complications due to the extensive myocardial injury. The patient experienced a favorable clinical outcome with complete recovery of left ventricular function and resolution of mitral insufficiency. This case exemplifies the necessity for the prompt identification of critical clinical manifestations and the initiation of treatment in accordance with established medical guidelines.

## Conclusions

This case report on TCM underscores the condition's complex nature and critical management aspects. TCM, often stress-induced and predominantly affecting women, manifests as a sudden, reversible left ventricular dysfunction. Key findings include the impact of drug-induced physical stress, such as serotonin syndrome in the patient discussed, which can exacerbate cardiovascular risks leading to TCM. The report highlights the diagnostic challenges, noting that TCM is usually identified during ACS assessments when no coronary obstruction is found. TCM involves an excessive release of catecholamines, leading to microvascular perfusion impairment, myocardial inflammation, and electrophysiological changes. The presence of QRS-ST-T waveform ST elevation is associated with cardiogenic shock, life-threatening arrhythmias, and a worse prognosis. This ECG pattern may arise regardless of a culprit coronary artery if a significant burden of the myocardium is acutely injured. The onset of QRS-ST-T wave fusion ST elevation can be considered a marker of impending hemodynamic instability. The report also delves into the various TCM phenotypes, treatment approaches, and the importance of vigilant monitoring and supportive care. It emphasizes that TCM's management is multifaceted, involving careful diagnostic evaluation and tailored therapeutic strategies to address the unique presentations and risks associated with the condition.

## References

[1] Boyd B, Solh T (2020). Takotsubo cardiomyopathy: review of broken heart syndrome. JAAPA.

[2] Matta AG, Carrié D (2023). Epidemiology, pathophysiology, diagnosis, and principles of management of Takotsubo cardiomyopathy: a review. Med Sci Monit.

[3] Goh AC, Wong S, Zaroff JG, Shafaee N, Lundstrom RJ (2016). Comparing anxiety and depression in patients with Takotsubo stress cardiomyopathy to those with acute coronary syndrome. J Cardiopulm Rehabil Prev.

[4] Pilgrim TM, Wyss TR (2008). Takotsubo cardiomyopathy or transient left ventricular apical ballooning syndrome: a systematic review. Int J Cardiol.

[5] Scantlebury DC, Prasad A (2014). Diagnosis of Takotsubo cardiomyopathy. Circ J.

[6] Ghadri JR, Cammann VL, Jurisic S (2017). A novel clinical score (InterTAK Diagnostic Score) to differentiate Takotsubo syndrome from acute coronary syndrome: results from the International Takotsubo Registry. Eur J Heart Fail.

[7] Samul-Jastrzębska J, Roik M, Wretowski D (2021). Evaluation of the InterTAK Diagnostic Score in differentiating Takotsubo syndrome from acute coronary syndrome. A single center experience. Cardiol J.

[8] Ghadri JR, Cammann VL, Napp LC (2016). Differences in the clinical profile and outcomes of typical and atypical Takotsubo Syndrome: data from the International Takotsubo Registry. JAMA Cardiol.

[9] Templin C, Ghadri JR, Diekmann J (2015). Clinical features and outcomes of Takotsubo (stress) cardiomyopathy. N Engl J Med.

[10] Ramaraj R, Sorrell VL, Movahed MR (2009). Levels of troponin release can aid in the early exclusion of stress-induced (takotsubo) cardiomyopathy. Exp Clin Cardiol.

[11] Kato K, Kitahara H, Fujimoto Y, Sakai Y, Ishibashi I, Himi T, Kobayashi Y (2016). Prevalence and clinical features of focal Takotsubo cardiomyopathy. Circ J.

[12] Citro R, Bossone E, Parodi G (2016). Independent impact of RV involvement on in-hospital outcome of patients with Takotsubo syndrome. JACC Cardiovasc Imaging.

[13] Cipriani A, D'Amico G, Brunello G (2018). The electrocardiographic "triangular QRS-ST-T waveform" pattern in patients with ST-segment elevation myocardial infarction: Incidence, pathophysiology and clinical implications. J Electrocardiol.

[14] Schreiber A, Inciong K, Ji W (2022). A single-center retrospective study on the incidence and clinical significance of the electrocardiographic "Triangular QRS-ST-T Waveform" pattern*. Heart Lung.

[15] Tarantino N, Santoro F, Guastafierro F (2018). "Lambda-wave" ST-elevation is associated with severe prognosis in stress (Takotsubo) cardiomyopathy. Ann Noninvasive Electrocardiol.

[16] Tarantino N, Santoro F, Brunetti ND (2018). Triangular "shark fin-like" ST modification in Takotsubo syndrome: challenging the concept of ST-elevation patterns without coronary occlusion?. J Electrocardiol.

[17] Ghadri JR, Wittstein IS, Prasad A (2018). International Expert Consensus Document on Takotsubo syndrome (part II): diagnostic workup, outcome, and management. Eur Heart J.

[18] Brown KH, Trohman RG, Madias C (2015). Arrhythmias in takotsubo cardiomyopathy. Card Electrophysiol Clin.

[19] Pant S, Deshmukh A, Mehta K (2013). Burden of arrhythmias in patients with Takotsubo cardiomyopathy (apical ballooning syndrome). Int J Cardiol.

[20] Medina de Chazal H, Del Buono MG, Keyser-Marcus L, Ma L, Moeller FG, Berrocal D, Abbate A (2018). Stress cardiomyopathy diagnosis and treatment: JACC state-of-the-art review. J Am Coll Cardiol.

